# Patient satisfaction – results of cluster analysis of finnish patients

**DOI:** 10.1186/s12913-023-09625-y

**Published:** 2023-06-13

**Authors:** Anu Nurmeksela, Markus Kulmala, Tarja Kvist

**Affiliations:** 1 Faculty of Health Sciences, Department of Nursing Science, University Lecturer University of Eastern Finland, P.O. Box 1627, Kuopio, 70211 Finland; 2 Faculty of Sport and Health Sciences, University of Jyväskylä, Research Centre for Health Promotion, P.O. Box 35, Jyväskylä, FI-40014 Finland; 3 Department of Nursing Science, University of Eastern Finland, Kuopio Campus, P.O. Box 1627, Kuopio, 70211 Finland

**Keywords:** Patient satisfaction, Quality of care, Nursing, Cluster analysis, Revised Humane Caring Scale, Questionnaire

## Abstract

**Background:**

Healthcare providers must understand patients’ expectations and perceptions of the care they receive to provide high-quality care. The purpose of this study is to identify and analyse different clusters of patient satisfaction with the quality of care at Finnish acute care hospitals.

**Methods:**

A cross-sectional design was applied. The data were collected in 2017 from three Finnish acute care hospitals with the Revised Humane Caring Scale (RHCS) as a paper questionnaire, including six background questions and six subscales. The *k*-means clustering method was used to define and analyse clusters in the data. The unit of analysis was a health system encompassing inpatients and outpatients. Clusters revealed the common characteristics shared by the different groups of patients.

**Results:**

A total of 1810 patients participated in the study. Patient satisfaction was categorised into four groups: dissatisfied (*n* = 58), moderately dissatisfied (*n* = 249), moderately satisfied (*n* = 608), and satisfied (*n* = 895). The scores for each subscale were significantly above average in the satisfied patient group. The dissatisfied and moderately dissatisfied patient groups reported scores for all six subscales that were clearly below the average value. The groups significantly differed in terms of hospital admission (*p* = .013) and living situation (*p* = .009). Patients representing the dissatisfied and moderately dissatisfied groups were acutely admitted more frequently than patients in other groups and were more likely to live alone than satisfied and moderately satisfied patients.

**Conclusion:**

The results mostly demonstrated high levels of patient satisfaction; however, the perceptions of minority populations of dissatisfied patients should be assessed to identify shortcomings in the care provided. More attention should be paid to acutely admitted patients who are living alone and the pain and apprehension management of all patients.

## Background

Promoting more patient-centred care has become a growing priority across European countries in recent years, aiming to enhance the quality of care and meet patients’ expectations. On average, patients in Finland have expressed greater satisfaction with the quality of care they receive compared to patients in other European countries [1]. To further improve patient-centred care, Finland has established a national nursing benchmarking network that currently includes twelve acute care hospitals. Patient satisfaction has been defined as one of the national nursing-sensitive quality indicators [2]. According to previous studies, Finnish patients have been quite satisfied with the quality of nursing care [3, 4]; however, there is also some evidence of decreasing trends in patient satisfaction [5]. To increase both their attractiveness to patients and patient involvement, some hospitals have established patient panels whose members collaborate with their staff. In these panels, patients have the opportunity to participate in developing a patient-oriented care culture by providing feedback on the quality of services, making suggestions to improve services, and bringing ideas to the design and development of services [6, 7]. It is important to identify groups of patients who are not satisfied with their care. In this study, we were interested in finding different patient satisfaction group clusters.

According to the World Health Organization (WHO), “quality of care is the degree to which health services for individuals and populations increase the likelihood of desired health outcomes” [8]. Care quality is linked to evidence-based professional knowledge, humane care, and avoiding harm. In this way, patient care is guided by a people-centred approach that responds to individual preferences, needs, and values. Furthermore, the quality of care is related to the quality of a healthcare system. This can be expanded to include the provision of timely, equitable, integrated, and efficient services regardless of gender, ethnicity, geographic location, and socio-economic status [8]. The importance of patients’ reported experiences of outcomes in enhancing patient-centeredness has been acknowledged, and it reflects all healthcare decision-making, processes, and care [9].

Previous studies have shown that patients appreciate effective and continuous interaction and communication with healthcare professionals (HCPs) and that these factors influence patients’ satisfaction, length of hospital stay, and recovery. The communication skills of HCPs are pivotal to ensuring that patients feel valued and cared for, as patients respond to how respectfully they are treated [10]. Patients are satisfied when they perceive that they are receiving personalised care [11], are respected, and are treated in a humane and caring environment [12]. Information provision and patient counselling have also been shown to be crucial for patient satisfaction [10]. Professionals should enable and support patients to become involved in the planning and decision-making related to their own care [13]. Patient satisfaction is also linked to patients’ confidence in treatment decisions [11]. The involvement of patients and their family members in care has been found to improve patient satisfaction outcomes through the reduction of adverse events, increased patient safety, and decreased length of hospitalisation [14].

Patient satisfaction is positively related to access to services [11], with waiting times for admission, interruptions in data flow [15], and inadequate pain management among the reasons for dissatisfaction [16]. Factors related to nurses, that is, poor work environment and job dissatisfaction, have been reported to negatively affect patient outcomes, such as increased complications and adverse events [17, 18], with heavy workloads among nurses associated with decreasing patient satisfaction [19]. Didier et al. (2020) suggested that interprofessional collaboration positively influences patient care, safety, and well-being [20], while the presence of highly educated nurses has been linked to high levels of patient satisfaction [21, 22]. Nursing management has been shown to both directly and indirectly affect patient outcomes and satisfaction [23, 24]; for example, nurses’ job satisfaction improves patient satisfaction with care [25].

To provide high-quality patient care, healthcare organisations must determine which aspects of care delivery are at subpar levels and require improvement. In this study, we examine patient satisfaction at three Finnish acute care hospitals. The purpose of this study is to identify and analyse different clusters of patient satisfaction with the quality of care at Finnish acute care hospitals.

## Methods

### Study design and participants

This study used a cross-sectional design. The data were collected in 2017 from three Finnish acute care hospitals. All hospitals were central hospitals with 425 to 513 beds and 2,388 to 2,880 employees [26]. The hospitals were in different parts of Finland. The study used convenience sampling of patients from each unit; patients from the inpatient wards (overnight hospitalisation) and outpatient departments (day procedures or treatments with same-day discharge) were invited to take part in the study. The data were collected through a paper questionnaire. A total of 50 questionnaires at each ward or department were distributed by nurses at the time when the patient (*n* = 3,050) was discharged from the hospital. The inclusion criteria were as follows: adult patients discharged from an inpatient ward or outpatient department who were capable of answering the questionnaire independently. Exclusion criteria included child patients as well as patients in the intensive care unit or operating room.

### Instrument

The Revised Humane Caring Scale (RHCS), administered via a paper questionnaire, was used to evaluate patient satisfaction with care [3, 27]. The instrument includes six background questions and six subscales:

1. *Professional practice* (17 items, e.g., “I was appreciated”, “The nursing staff were professional”);
2. *Information and participation in own care* (11 items, e.g., “I received enough information about my illness”, “I was able to participate in the planning of my care”);
3. *Human resources* (3 items, e.g., “The staff had enough time for me”);
4. *Pain and apprehension* (4 items, e.g., “I received medication for my pain at the right time”);
5. *Interdisciplinary collaboration* (3 items, e.g., “There was good collaboration between members of staff”); and.
6. *Outcome variables* (4 items, e.g., “I set a clear goal for my care together with the staff”).

The subscales include a total of 42 items, which respondents graded from 0 to 10, with the response options ranging from *totally disagree* (0) to *totally agree* [10]. Cronbach’s alpha values between 0.775 and 0.970 have been reported for the RHCS [3, 27, 28].

### Data analysis

The *k*-means cluster analysis was used to identify different groups of patients based on their evaluations of their satisfaction with care. Clusters were used to describe the characteristics shared by the groups in question. In this study, the unit of analysis was a health system encompassing inpatients and outpatients, as the profiles of the hospitals were sufficiently similar. Firstly, the participants’ percentages of the given background variables were reported. Then, *k*-means clustering [29] analysis was performed using the statistical software R [30]. This is a widely used clustering algorithm that classifies data into *k* distinct groups, or clusters, based on a predetermined value for *k*. In this study, the optimal value for *k* (*k* = 4) was chosen based on an elbow plot with *k* values ranging from 1 to 10. The algorithm is designed to provide clusters that are internally as similar as possible while exhibiting low inter-cluster similarity. After the analysis, information regarding the background variables of the four clusters was reported. This allowed comparisons of the relationships between clusters and background variables using the χ2 (Chi-square) test.

## Results

A total of 1810 patients participated in the study. The response rate was 59%. Of these participants, 75% were female, and the majority lived with a spouse or other person (74%). Half of the participants had a vocational degree, while 26% had a university degree. Concerning occupation, over half of the participants were not actively working, which meant that they were either pensioners, students, or unemployed, while 20% of the participants were employed and 23% held the position of official. Of the participants, one-third were acutely admitted to the hospital, while most of the patients (67%) were admitted as planned. The most common reasons for hospitalisation were treatment (64%) and examination (22%) (Table 1).

**Table 1:** Background information for the groups of dissatisfied, moderately dissatisfied, moderately satisfied, and satisfied patients (*n*, %, χ^2^, *df*, *p*)

(n)	Dissatisfied patients n (%) 58 (3)	Moderately dissatisfied patients n (%) 249 (14)	Moderately satisfied patients n (%) 608 (34)	Satisfied patients n (%) 895 (49)	χ^2^	df		*p*	
Gender					3.7068	3	0.295		
Male (405)	19 (33)	91 (37)	253 (42)	42(38)					
Female (1247)	39(67)	158 (63)	352 (58)	548 (62)					
Education					5.1288	6	0.527		
Vocational degree (884)	23 (42)	122 (50)	306 (51)	433 (49)					
No education/ other education (437)	20 (36)	62 (25)	145 (24)	210 (24)					
University degree (454)	12 (22)	60 (25)	149 (25)	233 (27)					
Occupation					9.9291	6	0.128		
Not actively work (1024) *Pensioners (884)* *Students (46)* *Unemployed (43)* *Other (51)*	30 (53)	125 (50)	345 (57)	524 (59)					
Employed (356)	11 (20)	51 (21)	131 (22)	163 (18)					
Official (420)	15 (27)	73 (29)	130 (21)	202 (23)					
Admission to hospital					10.846	3	0.013*		
Acute (618)	25 (43)	88 (35)	231 (38)	274 (31)					
Planned (1280)	133 (57)	161 (65)	373 (62)	613 (69)					
Reason for hospitalisation					3.614	6	0.729		
Other (227)	10 (17)	29 (12)	72 (12)	116 (13)					
Treatment (1063)	40 (69)	16 (67)	416 (69)	591 (66)					
Examination (362)	8 (14)	52 (21)	116 (19)	186 (21)					
Living status					11.634	3	0.009*		
With a spouse or other (1298)	34 (59)	176 (71)	450 (75)	683 (76)					
Single (453)	24 (41)	71 (29)	152 (25)	206 (23)					

Significance: * = *p* < .05

The cluster analysis classified the patients into four groups: dissatisfied patients (*n* = 58), moderately dissatisfied patients (*n* = 249), moderately satisfied patients (*n* = 608), and satisfied patients (*n* = 895) (Table 2). The optimal number of clusters [4] was determined by examining an elbow plot containing 1 to 10 clusters.

Members of the dissatisfied patient group were mostly female (67%), not actively working (53%), admitted to the hospital as planned (57%), and living with a spouse or other person (59%); the most common educational qualification in this group was a vocational degree (42%). There were only 58 respondents in this group, which represented 3% of the participants; as such, this was the smallest group identified through cluster analysis (Table 1).

The moderately dissatisfied patient group consisted mostly of females (63%) who held a vocational degree (50%). In addition, most of the patients in this group were not actively working (50%), were admitted to the hospital as planned (65%), were hospitalised for treatment (67%), and lived with a spouse or other person (71%). This group had 249 respondents, which reflected 14% of all participants (Table 1). Patients in the moderately dissatisfied group were less satisfied with all subscales of care when compared to the moderately satisfied group. The *Pain and apprehension* and *Outcome variables* subscales received the lowest scores from this group of patients, with scores well below the average (Table 2).

**Table 2:** Min, Median, Mean, Max and **S**tandardised mean by the groups of dissatisfied, satisfied, moderate satisfied, and moderate dissatisfied patients

	Dissatisfied patients (n = 58, 3%)	Moderately dissatisfied patients (n = 249, 14%)	Moderately satisfied patients (n = 608, 34%)	Satisfied patients (n = 895, 49%)
Professional practice				
Min	1.000	5.529	7.176	8.294
Median	5.588	7.941	9.000	9.941
Mean	5.158	7.887	9.014	9.846
Max	7.941	9.765	10.000	10.000
Standardised mean	-3.5674507	-1.1270376	-0.1187430	0.6254081
Information and participation in own care				
Min	0.6364	2.091	4.000	7.818
Median	4.6818	7.273	8.800	9.909
Mean	4.2593	7.180	8.671	9.724
Max	7.2727	9.364	10.000	10.000
Standardised mean	-3.3252672	-1.2073664	-0.1261241	0.6370762
Human resources				
Min	0.000	0.000	0.000	5.333
Median	3.833	7.000	8.333	10.000
Mean	3.236	6.553	8.028	9.592
Max	7.000	10.000	10.000	10.000
Standardised mean	-2.7328858	-0.9924812	-0.2187180	0.6018053
Pain and apprehension				
Min	0.500	0.000	0.000	4.750
Median	3.750	6.250	8.500	10.000
Mean	3.443	5.909	8.106	9.484
Max	8.000	9.000	10.000	10.000
Standardised mean	-2.4921544	-1.2358133	-0.1169114	0.5847426
Interdisciplinary collaboration				
Min	0.3333	3.000	5.000	7.333
Median	5.3333	8.000	9.000	10.000
Mean	5.2931	7.813	8.943	9.882
Max	10.0000	10.000	10.000	10.0000
Standardised mean	-3.1766974	-1.0933438	-0.1583924	0.6176465
Outcome variables				
Min	0.000	1.000	2.500	7.000
Median	4.000	7.000	9.000	10.000
Mean	3.743	6.861	8.828	9.788
Max	7.000	10.000	10.000	10.000
Standardised mean	-3.18911771	-1.24945506	-0.02549518	0.57160246

A comparison of the two smallest groups (dissatisfied and moderately dissatisfied patients) showed that the mean values for patient satisfaction were at poor or moderate levels. In the dissatisfied patient group, the mean values for all of the subscales were between 3.236 and 5.2931, while patients in the moderately dissatisfied group scored their satisfaction with care between 5.909 and 7.813; all of these assessments fell below the average value (Table 2). Comparisons of the profiles of the identified groups revealed clear differences in hospital admission and living status. More specifically, the dissatisfied and moderately dissatisfied patients were more often acutely admitted to the hospital than satisfied patients and more likely to live on their own than satisfied or moderately satisfied patients (Table 1).

Members of the moderately satisfied patient group were mostly female (58%), held a vocational degree (51%), were not actively working (57%), were admitted as planned (62%), were hospitalised for treatment (69%), and lived with a spouse or other person (75%). This group included 608 respondents (34%) and was the second largest group identified through cluster analysis (Table 1). The score for the *Outcome variables* subscale for the group of moderately satisfied patients was closest to the mean value but also fell below it (Table 2).

The satisfied patient group consisted mostly of female patients (62%), individuals who were not actively working (59%), were admitted as planned (69%), were hospitalised for treatment (66%), and lived with a spouse or other person (76%); most of the members of this group held a vocational degree (49%). This group included 895 participants (49%) and represented the largest cluster (Table 1). When the two largest groups (satisfied and moderately satisfied patients) were compared in terms of mean values for various satisfaction subscales, both groups assessed satisfaction as being at an excellent or good level. In the satisfied patient group, the mean values for all subscales were between 9.484 and 9.882, which can be compared to 8.028 and 9.014 in the moderately satisfied patient group. However, it is important to state that while the satisfied patient group assessed patient satisfaction above the average value, the moderately satisfied patients’ assessments of patient satisfaction fell below the average value (Table 2). In terms of the profiles of the identified groups, the satisfied patient group showed larger differences in gender than the moderately satisfied patient group (Table 1).

There were no statistically significant between-group differences in gender, education, occupation, or the reason for hospitalisation. In contrast, the four groups demonstrated significant differences in the patients’ admission to the hospital (*p* = .013). For example, the satisfied patient group had relatively more patients who were admitted to the hospital as planned than the dissatisfied patient group, which had a higher share of acutely admitted patients than other groups. There were also statistically significant between-group differences for living status (*p* = .009). The satisfied patient group had a relatively higher share of respondents who lived with a spouse or other person than the dissatisfied patient group (Table 1).

## Discussion

The purpose of this study was to identify and analyse different clusters of patient satisfaction with the quality of care at Finnish acute care hospitals. As patient satisfaction is a widely studied topic in health and nursing research, we wanted to look at the data using cluster analysis, which is not a commonly used analysis method, and produce some novel findings in this study.

Results of the cluster analysis showed that patients in the most satisfied group were consistently satisfied with all measured aspects of their care. Overall, 83% of patients were satisfied or moderately satisfied. Similar findings with similar sampling strategies were reported when the RHCS instrument was used to measure patient satisfaction with the quality of care in Finland [3, 4, 31] and other countries [32, 33]. However, other studies have also shown that nursing workload management is associated with patient satisfaction and can influence the patient experience [23], especially respect, communication, and patient involvement in the planning of care [19]. In addition, other researchers have provided evidence for the link between nurses’ job satisfaction and patient satisfaction [17, 18, 24, 25]. Therefore, organisational factors, such as the management of nurse managers’ work activities (team or dual model), relational leadership styles, and patient-centred values, can influence patient outcomes and satisfaction [18, 24, 28, 34]. Many organisations have also developed various models to improve patient-centred care. The trend and objective are to involve patients in quality-improvement committees [35] or hospitals’ customer panels [6, 7].

The dissatisfied patients’ group only included 3% of all patients, while the moderately dissatisfied patient group included 14% of the participants. Both groups were most dissatisfied with the *Human resources*, *Pain and apprehension*, and *Outcome variables* subscales in particular. Previous study findings show that heavy workloads among nurses can decrease patient satisfaction [19], and a lower nurse-patient ratio can adversely affect the quality of care and patient safety [36]. Previous studies have also found that patients perceive a need for better pain management and are dissatisfied with the limited information they receive during patient education, treatment planning, and pain management [37]. A big challenge for health organisations is to maintain excellent quality of care. Simultaneously, the shortage of nurses is worsening, and more attention should be paid to developing patient-centred processes.

According to the results, patients who were admitted to the hospital as planned were more satisfied with the care they received than patients who were acutely admitted. This is not surprising, as other studies have identified many challenges that are present among acutely admitted patients. For example, rush and waiting times in emergency care, the heavy workload of professionals, and poor interactions between HCPs and patients are challenges associated with acute care [15, 38]. This is because patients have certain expectations regarding the level of professionalism they receive during their time in the emergency room or other healthcare services. This includes asking how they are feeling, whether they are in pain, and ensuring they are not left unattended. Therefore, acutely admitted patients are a clear priority for care, and it is necessary to develop interventions that cater to their specific expectations.

The patients who participated in this study evaluated interdisciplinary collaboration as being at a relatively good level. The results also revealed acceptable patient satisfaction with overall care, with involvement from various HCPs. In other words, patients did not differentiate between physicians, nursing staff, or other practitioners when evaluating the care provided [39]. This was particularly evident when patients were asked about their satisfaction with the staff in the care unit. The only exception was when they were asked separately about their satisfaction with nurses’ and doctors’ professional skills. Therefore, it is important to remember that the care process is a joint, interprofessional responsibility and that multi-professional teams should plan patient care together with patients and their close relatives.

The presented findings showed that patients who lived with their spouse or someone else were generally more satisfied with care than patients who lived alone. Family involvement in patient care has been found to increase patient satisfaction, particularly in situations related to patient education and discharge planning [40]. In addition, Giap and Park (2021) found that interventions that promote patient and family involvement can reduce the prevalence of adverse events, decrease the length of a hospital stay, improve patient perceptions of safety, and improve patient satisfaction [14].

Most of the respondents (75%) were female, which could indicate that women were more willing to give their opinion about care than men. In the study by Ek (2013), women demonstrated greater interest in and actively sought out more health-related information than men [41]. This may explain the higher level of active engagement observed among women in this study. It’s noteworthy that a total of 83% of all patients were satisfied with their care. It is also possible that the study did not reach patients who were not satisfied or that they were among the 41% of individuals who did not answer the questionnaire. In the future, it is important to consider research strategies that ensure the voices of all patients are heard. By involving patients in quality-improvement committees in collaboration with HCPs, healthcare organisations can foster a patient-centred culture and improve patient satisfaction and care quality.

### Strengths and limitations

Regarding the strengths of this study, the RHCS is a reliable, validated instrument for measuring patient satisfaction. It has been widely used in research covering numerous countries [3, 19, 27, 28, 32, 33]. Moreover, the size of the study population was another strength, as 1,810 patients participated in the research, representing 59% of those invited to the study. However, it is important to acknowledge that the generalisability of the results within Finland may be limited due to the convenience sampling method employed. For example, further research in other European countries is needed to obtain more comprehensive international coverage and comparable data. Patients’ anonymity was protected in various ways to encourage confident and truthful responses. Patients had the option to complete the questionnaire either at the hospital before discharge or at home. In both cases, they returned the questionnaire in a sealed envelope addressed to the researcher. Along with the questionnaire, patients received an information sheet concerning the research, where they were informed that only the research team processes the data and that responses are reported anonymously. No identifying information about patients was collected. The study also has some limitations. Firstly, the data set includes all the responses, even if a respondent did not answer certain items. It is important to note that empty items were not included in the analysis. The data were collected from both inpatient wards and outpatient departments, which may have introduced variations in the quality of care evaluations, particularly considering that the amount of time patients spend with HCPs is typically limited in outpatient settings. We did not collect the patients’ ages, a decision that may have affected the results of the cluster analysis; nevertheless, all of the patients were adults.

## Conclusion

Patients reported high levels of satisfaction with the quality of care, even though there were still minority groups that showed satisfaction levels that fell far below the average value; these groups must be analysed to identify shortcomings in care quality. For example, more attention should be paid to acutely admitted patients who are living on their own, as well as pain and apprehension management for all patients.

## Data Availability

All of the data supporting our findings are presented within the manuscript.

## Abbreviations

df: Degrees of Freedom
HCPs: Healthcare Professionals
n: Number of Participants
p: Significance
RHCS: Revised Humane Caring Scale
χ: Chi-Square Test
